# A Successful Bisphosphonates Monotherapy in Spinal Form of Paediatric Chronic Recurrent Multifocal Osteomyelitis (CRMO)—Case Report

**DOI:** 10.3390/metabo13030344

**Published:** 2023-02-25

**Authors:** Aleksandra Opala, Jagoda Hofman, Michał Hutny, Aleksandra Wylazlowska, Paweł Matusik

**Affiliations:** 1 Department of Paediatrics, Paediatric Obesity and Metabolic Bone Diseases, Faculty of Medical Sciences in Katowice, Medical University of Silesia, 40-055 Katowice, Poland; 2 Scientific Society of Medical Students, Faculty of Medical Sciences in Katowice, Medical University of Silesia, 40-055 Katowice, Poland; 3 Department of Paediatrics, Paediatric Obesity and Metabolic Bone Diseases, Municipal Hospital, 43-100 Tychy, Poland

**Keywords:** chronic recurrent multifocal osteomyelitis, bisphosphonates, pamidronate, children

## Abstract

Chronic recurrent multifocal osteomyelitis (CRMO) is a non-infectious inflammatory disorder resulting from the multifocal bone and bone marrow lesions with periodic relapses and remissions and with an uncertain prognosis. Treatment options in CRMO are based on expert opinion and relatively small groups of patients. A nine-year-old female patient with no significant past medical history presented with compression fractures and multifocal bone lesions in the thoracic and lumbar spine, as shown in imaging (CT, MRI). Densitometry revealed a diffuse decrease in bone density. Based on the patient’s clinical image and above examinations, the other possible aetiologies—infectious (including tuberculosis), neoplasms, Langerhans cell histiocytosis—were ruled out, which led to eventual final diagnosis—CRMO. The patient was successfully treated with pamidronate infusion initiated in cycles over three consecutive days every 3 months. In addition to clinical improvement, there was a significant remission of inflammation and bone structure healing assessed by MRI after four treatment cycles. Intravenous bisphosphonates usage seems to be a good therapeutic option in CRMO paediatric patients with spinal localization of the lesions complicated by compressive fractures. However, more data, based on larger patient populations, are needed to provide a detailed paediatric CRMO treatment algorithm.

## 1. Introduction

Bone pain is a common symptom in children, which may result from physiological outgrowth, but also be a consequence of pathological processes present in the organism. One of the potential causes may be chronic recurrent multifocal osteomyelitis (CRMO), an autoimmune inflammation resulting from an imbalanced immune response, consisting of overexpression of cytokines by immune cells, including monocytes [1]. This disorder is the most severe form of chronic non-bacterial osteomyelitis (CNO) [1]. Its clinical presentation is characterised by multiple focal aseptic lesions, found predominantly in metaphyses of long bones [2], which exert typical characteristics of inflammatory bone pain—nocturnal intensity peak, wearing-off associated with physical activity, comorbid tissue sclerosis [3]. Due to a rather non-specific pain symptomatic, the differential diagnosis of this disease includes a wide range of disorders presenting with similar symptoms: infectious osteomyelitis, septic arthritis, osteonecrosis, psoriatic arthritis, as well as neoplasms (leukaemia, lymphomas, Langerhans cell histiocytosis, primary malignant bone disease) [4]. Other features multiplying potential differential diagnoses are vertebral compression fractures and collapse, typically associated with spinal presentation of this disorder [5]. The same alterations might be observed in musculoskeletal tuberculosis [6] and spinal-localised neoplastic entities [7]. In order to diagnose a patient with CRMO, the above conditions must be taken into consideration and excluded, based on the patient’s clinical image, biomarkers assessment and imaging methods [8].

The population affected with CRMO to the highest extent are children and adolescents (age group: 2 to 17 years; mean onset: 10 years) [3], with a prominent predominance found in females (4 × higher prevalence than in males). Despite a reasonable number of cases reported in the literature, this disorder is considered rare, as its general prevalence ranges from 1:160,000 to 1:2,000,000 [2]. The lesions are located most frequently in lower extremities (ca. 20–50% of lesions), especially in the pelvis [9,10]. Spinal lesions are found less frequently, amounting to ca. 13–17% of lesions [9,10]. It is the latter localisation which is associated with compressive fractures and malformations, leading to the developmental problems [11]. As the main pathophysiological factor causing the CRMO is the autoinflammatory process, non-steroid anti-inflammatory drugs (NSAIDs) are a natural first-line treatment strategy [12], which enables an efficient disease management in most cases [13]. Nevertheless, there are cases, in which NSAIDs do not reverse the disorder progression—in these patients the second-line treatment may provide the optimal control of the pathology. Therapy strategies include bisphosphonates, as well as biological medication (IL-1 blockers, IL-23 and IL-17 inhibitors) [8].

In accordance with CARE Case Report Guidelines [14,15], the current case report presents a pre-teen girl with a rarely found spinal form of CRMO, which was successfully controlled using solely pamidronate, further evidencing the efficacy of bisphosphonate monotherapy in this disease. The patient’s symptomatology is an example of postural defects, which may develop as a complication in a course of CRMO affecting the vertebrae. This report also presents an efficient diagnostic assessment methodology, which encompasses not only the diagnostic process of the CRMO itself, but also the less frequent features of this disease, including osteoporosis, which in the current patient was adequately treated with pamidronate.

## 2. Case Report

### 2.1. Patient Information

A nine-years-old female patient with no significant past medical history presented with compression fractures and multifocal bone lesions in the thoracic and lumbar spine. The patient reported pain in the lumbar area which emerged after minor trauma. The pain increased in time and was triggered by movement lamination and flexion position. The patient was initially treated with non-steroid anti-inflammatory drugs (NSAIDs) and her thoracic and lumbar spine was supported by Jewett brace.

### 2.2. Clinical Findings

**Figure 1:**
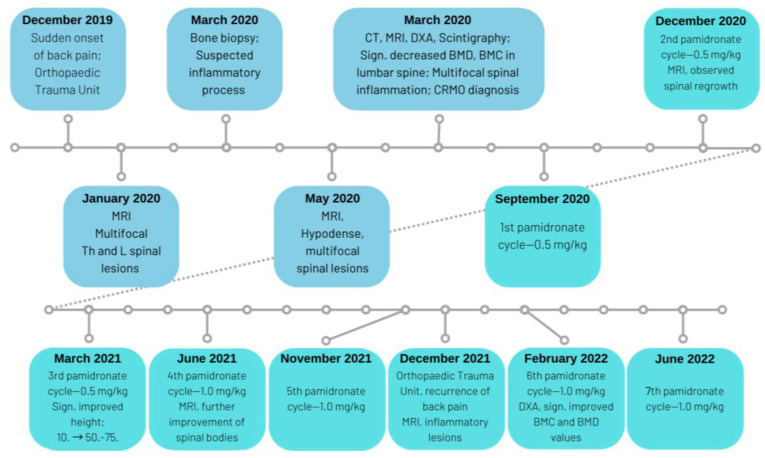
The timeline of patient’s diagnostics and treatment.

In the physical examination. a series of abnormalities were found, which all could be considered secondary to the patient’s vertebral morphology disorders. The patient’s body-mass index [BMI] values (22.84 kg/m^2^) laid within the range allowing classification as overweight. Her torso was shortened, as observed in a sitting position. Moreover, her left lower extremity was also asymmetrically shorter than the contralateral leg. A pathological thoracic kyphosis was observed, accompanied by shoulder asymmetry. The timeline of diagnostic assessments, as well as therapeutic interventions, are described in further sections of the article and summarised graphically below in Figure 1.

### 2.3. Diagnostic Assessment

Time from the first symptoms onset to diagnosis was 8 months. The conditions considered to be plausible explanations of the symptomatology, i.e., infections (including tuberculosis), neoplastic process, Langerhans cell histiocytosis (LCH), connective tissue diseases and metabolic disorders, were excluded in the process of consequent diagnostic assessment. Laboratory tests were provided for initial differential diagnoses. Blood morphology analysis did not show any significant abnormalities, renal and liver function tests were normal, as were C-reactive protein level, erythrocyte sedimentation rate, lactate dehydrogenase and serum electrolytes level. The blood culture for aerobic bacteria was negative. The relevant observations were an elevated level of total serum IgG, which could indicate a chronic inflammatory response, as well as an elevated total serum protein concentration. The biochemical markers of calcium-phosphate metabolism were within the reference range. The serum level of alkaline phosphatase was normal. The QuantiFERON test was provided to exclude tuberculosis. Autoantibodies characteristic for rheumatic disorders were negative. The two bone biopsies were performed, leading to negative cultures. Histopathological examination revealed a non-specific inflammatory process, though no neoplastic cells were observed. For exclusion of LCH, the immunohistochemical evaluation of bone marrow for CD1 and s100 expression was conducted.

**Figure 2:**
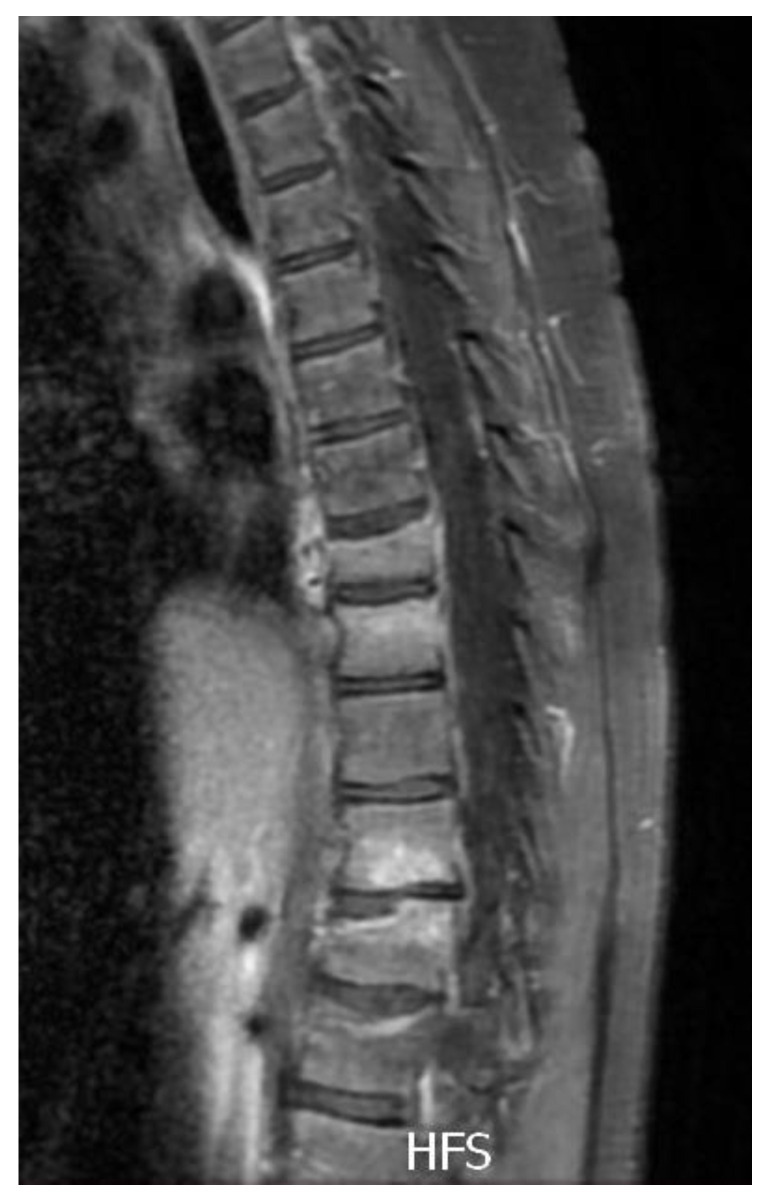
MRI of the spine depicting the vertebral lesions prior to the pamidronate treatment.

Imaging techniques such as magnetic resonance imaging (MRI) and computer tomography (CT) scans of thoracic and lumbar spine revealed compressive fracture of Th8, Th12 and L1 vertebral bodies, which were characterized by abnormality of trabecular structure and marginal ridges, which in the case of Th8 led to development of vertebra plana and multifocal lesions Th7–Th12 (Figure 2).

Further handling involved more advanced methods—dual-energy X-ray absorptiometry (densitometry; DXA) and scintigraphy. The results of DXA in the lumbar spine (L1–L4) as well as of the total body showed a significantly decreased bone density, z-score for lumbar spine (LS), and the total body less head (TBLH) measurement was—2.9. A summary of results of this examination is presented below in Table 1.

Densitometry was also repeated in February 2022 as a means of controlling the therapy with bisphosphonates. The patient’s results changed significantly within 1.5 years between the initial and repeated DXA, as in comparison to the above results the patient’s lumbar spine bone density z-score was 1.2, whereas the z-score for the total body equaled 0.9.

The scintigraphy was conducted using a technetium 99m-methyl diphosphonate (99mTc MDP) radiotracer. The radiotracer binding ratio was increased in multiple foci bilaterally in Th8–Th12 vertebral bodies, with a peak intensity in the Th10 level. Due to the suspected metaphyseal dysplasia and systemic osteoporosis, a roentgenography was performed, including posterior-anterior (PA) scans of the right hand and head. The bone tissue in the structures examined was physiological, with the exception of mild signs of metacarpal bones osteoporosis. Both clinical signs and the results of diagnostic procedures allowed CRMO to be diagnosed by exclusion.

### 2.4. Therapeutic Intervention

**Figure 3:**
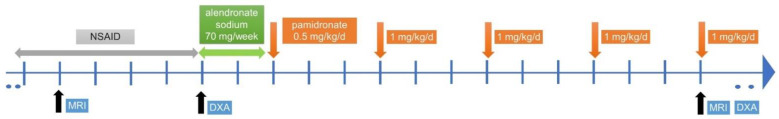
A timeline of therapeutic interventions (months). DXA, dual-energy X-ray absorptiometry; NSAID, non-steroid anti-inflammatory drug; MRI, magnetic resonance imaging.

Treatment with pamidronate infusion was initiated in cycles over three consecutive days every 3 months. Before the administration of the pamidronate, the patient was treated with an oral sodium alendronate dose of 70 mg per week for 3 consecutive months. The daily dosage of pamidronate was calculated in relation to the patient’s current weight using the formula: 0.5 mg/kg in the first cycle, and 1 mg/kg from the second. Prior to the pamidronate infusion, intravenous paracetamol was given for prevention of side effects such as fever and muscle ache. In addition to the pamidronate, supplementation of calcium, magnesium and vitamin D was implemented. The therapeutic process is summarised below in Figure 3.

### 2.5. Follow-Up and Outcomes

The effectiveness of therapy was verified by imagining techniques in addition to clinical improvement. Clinically, relief of pain and improvement of spine movability were observed. The patient tolerated treatment well, and no early and late onset complications were observed.

**Figure 4:**
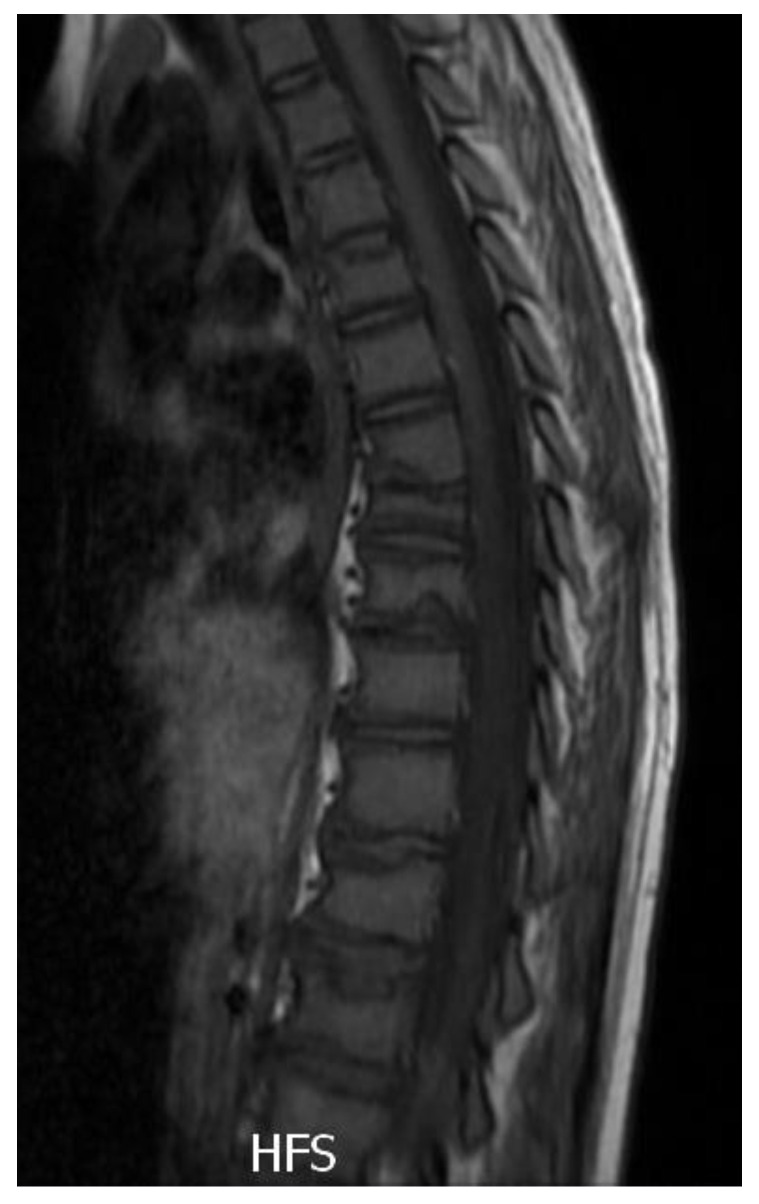
MRI of the spine after 1 year of pamidronate therapy (4th pamidronate cycle).

MRI and DXA were performed in order to assess the outcome of treatment. The MRI imagined the sclerotic process of fractured vertebral bodies. There was a significant remission of the inflammation and bone structure healing—no new active lesions were revealed (Figure 4).

The densitometry scans for TBLH and LS projections showed significant improvement in BMD. The course of patient’s clinical improvement is presented below in Table 2. in relation to the consecutive pamidronate cycles.

## 3. Discussion

Despite the growing body of evidence in the literature concerning the therapeutic strategies for CRMO, this rare disease remains a challenging issue without specific recommendations. Even among the relatively small population of patients affected by this disorder, there are some major differences in the clinical phenotype, as well as in response to treatment. Though the spinal manifestations are not atypical presentations of CRMO, they are less frequent than lesions found in long bones [16]. This phenotype is associated with deformities within the structure of vertebrae, which disrupt the kinetic balance of the vertebral column, leading consequently to further defects such as kyphosis [17], as was found in the case presented in this report. A comorbid issue found in the current patient was a severe osteoporosis, inappropriate for her age. It is not a common feature of CRMO, though as presented in this case report, it may successfully be controlled with bisphosphonate treatment of the main disorder.

Due to unspecific symptomatology, the diagnostic assessment of CRMO is a challenge requiring a multidisciplinary and multidirectional approach [18]. Though this disorder is considered rare, its actual prevalence might be higher than reported. It is thought to be underdiagnosed due to false identification as one of the numerous differential diagnoses of CRMO [19,20]. Though the genetic background of CRMO has not yet been precisely elucidated [21], the results of recent genetic studies showed significant differences in gene expression between patients with CRMO and otherwise healthy study participants [22]. As in cases of other syndromes associated with sterile osteomyelitis (Majeed, DIRA and PAPA syndromes), which were proven to be caused by single gene mutations [21], further analyses could lead to establishing reliable diagnostic tools for genetic assessment and evaluation of CRMO diagnosis [22]. The imaging technique, which in previous studies has been indicated as that offering the earliest and the best differentiating potential, consists of whole-body MRI scanning [10,23]. In the presented case, it was also MRI which led to an early discovery of multifocal lesions, and in consequence aroused the suspicion of CRMO.

One of the differential diagnoses is that for neoplastic disorders involving bone tissue, therefore scintigraphy using 99mTc MDP radiotracer is a valuable supplementary diagnostic method, which allows differentiation between the inflammatory processes, such as those found in CRMO, and neoplastic progression [24].

In cases of more severe CRMO, instead of first-line medicaments, NSAIDs or, in combination with them, a wide range of second-line treatment options is available, including disease-modifying anti-rheumatic drugs (DMARDs), glucocorticoids and bisphosphonates [25,26]. Pamidronate, classified as a nitrogen-containing bisphosphonate, inhibits farnesyl diphosphate synthase in osteoclasts by mimicking its substrate. This in turn prevents cell polarisation and adhesion due to decreased prenylation of small GTPases, which together with induction of osteoclast apoptosis ultimately decreases intensity of bone resorption [27]. This mechanism of action is advantageous in terms of CRMO pathophysiology, which consists of inflammatory induced bone resorption. Early reports suggest that the bisphosphonates offer promising efficacy in controlling CRMO, as well as safety of use [16,28], though results of recent studies indicated a burden of adverse events (AEs) in pamidronate therapy. In some patients, bisphosphonates were found to worsen bone pain [25], as well as lead to nausea, headache, fever and phlebitis [29], which ultimately decrease patients’ compliance [30]. The presented patient’s therapy was well tolerated and led to no significant AEs. As for therapeutic safety, its efficacy was also ambiguously discussed in recent studies. Some reports state that more than 2/3 patients treated with bisphosphonates achieved remission of CRMO symptoms and the progression of their disorder was inhibited, both in spinal and peripheral lesions [25,26,29,30,31,32,33,34]. It was also suggested that pamidronate leads to a rapid improvement in patient’s condition [26], which is in accordance with the observations of this study—within 1.5 years the reported patient returned to regular height range (50.-75. centile) from the threshold of 10. centile. It should also be noted, that pamidronate was used in this case as a monotherapy, as opposed to previous reports where the preferred method was combination with DMARDs or glucocorticoids [35]. The frequency of bisphosphonate infusions was in accordance with previously described therapeutic attempts, though the quantity of cycles exceeded the previously reported mean [32,35,36]. Nevertheless other studies presented cases in which pamidronate was inefficient in controlling CRMO [17,37,38]. The aetiology of differences in response to bisphosphonates treatment is not yet clear.

In conclusion, CRMO diagnosis has to be considered in every child with low-energy compressive fractures with recurrent pain. Intravenous bisphosphonates usage seems to be a good therapeutic option in CRMO paediatric patients with spinal localization of the lesions complicated by compressive fractures. However, more data, based on larger patient populations, are needed to provide a detailed paediatric CRMO treatment algorithm.

## Figures and Tables

**Table 1:** The results of DXA conduced on 13 July 2020.

ROI	BMD (g/cm^2^)	BMC (g)	Surface (cm^2^)	Z-Score
L1	0.317	1.83	5.76	−3.2 (−39%)
L2	0.355	2.81	7.91	−3.0 (−37%)
L3	0.351	2.29	6.53	−3.1 (−37%)
L4	0.404	2.93	7.25	−2.2 (−27%)
TBLH	0.359	9.86	27.46	−2.9 (−35%)

BMC, bone mineral content; BMD, bone mineral density; ROI, region of interest; TBLH, total body less head.

**Table 2:** Summary of patient’s course of treatment.

Date of Hospitalization (Cycle)	Weight in kg (Centile)	Height in cm (Centile)	Dose of Pamidronate (mg/kg)	Total Body bone Density z-Score
13 July 2020 (pre-treatment)	38.0 (75.-90.)	129.0 (10.)	-	−2.9
18–20 September 2020 (1st cycle)	-	-	0.5	-
18–20 December 2020 (2nd cycle)	35.7 (50.-75.)	137.1 (25.-50.)	0.5	-
19–21 March 2021 (3rd cycle)	36.3 (50.-75.)	142.0 (50.-75.)	0.5	-
25–27 June 2021 (4th cycle)	-	-	1.0	-
19–21 November 2021 (5th cycle)	39.9 (50.-75.)	147.0 (50.-75.)	1.0	-
11–13 February 2022 (6th cycle)	42.3 (50.-75.)	148.0 (50.-75.)	1.0	1.2
13–15 June 2022 (7th cycle)	46.1 (75.)	150.0 (50.)	1.0	-

Footnote: Onset of adolescence, defined as Stage ≥ 2 of Tanner Scale, was marked with double line between 1st and 2nd pamidronate treatment cycle.

## Data Availability

Data are available upon request. The data are not publicly available due to the privacy law regulations of the hospitals in which the patient was treated.
